# A New Adhesive for Traction

**Published:** 1918-09

**Authors:** 


**A New Adhesive for Traction.** W. F. CUNNINGHAM, 1st. Lieut., M. R. C. Preliminary Note of an article to appear elsewhere.

The material which has been used is a solution of celluloid in acetone. It is more reliable and efficient as an adhesive than those in vogue at the present time and is easily prepared. A particular advantage, however, is that the desired traction may be applied immediately.

For general purposes a solution of 7.5 g. of celluloid in 100 cc. of acetone is found to be the best. This percentage may be increased up to 10 g. per 100 cc., where only small areas of skin are available.

It is prepared by placing small pieces of celluloid scrap in a dry, clean, moderately wide mouthed bottle securely stoppered, and is shaken at frequent intervals. It makes a clear homogeneous, syrupy fluid and is ready for use in from 48 to 72 hours. Owing to the volatility of acetone, precautions should be taken to prevent evaporation. One liter is sufficient for at least 30 applications.

*Mode of Application.* No preparation of the skin is necessary; it should, however, be dry. Shaving should be avoided as the hairs increase its effectiveness. Iodine and also picric acid and alcohol should be avoided as far as possible on areas to which any type of adhesive is to be applied. Both of these drugs increase exfoliation and are often responsible for early slipping.

The solution is poured on to the skin and distributed with a small, rather stiff brush. Canton flannel strips are found to be the best material for traction bands. They should be applied *immediately* and smoothly and a thin coating of the solution used over them. A circular gauze bandage should then be applied.

Twenty-five applications have been carefully observed over periods of from 10 to 43 days. The substance itself is not irritating, but blistering may occur at the margins of the strips when excessive traction is used.

The following are a few examples of experiments:

1. Traction suspension apparatus for fracture of femur. Adhered area for strips $6'' \times 3''$. Thirty lbs. of traction applied as soon as apparatus could be set up, and this was just less than 10 minutes.

2. 10 o/o solution applied to dorsal and plantar surfaces of great toe as far as metacarpo-phalangeal joint, and the entire leg suspended by means of muslin bands.

3. With a 6 o/o solution applied to forearm, area 1 3/4″×6″ using Canton flannel strips, sufficient traction was exerted three minutes after application to pull patient and hospital bed, without castors, several feet and strips were still holding.

*Conclusions.*

1. 7.5 o/o celluloid in acetone makes an excellent adhesive material.
2. Its rapidly volatilizing quality permits of immediate traction.
3. It is insoluble in water — hence, not affected by perspiration or climatic changes.
4. Solutions which are likely to increase desquamation should not be used prior to application.
5. The natural process of exfoliation of epidermis limits the effectiveness of one application to about three weeks, though single application has adhered for 43 days.

*Note.* All experiments were done with combs, but through the kindness of the American Celluloid Company we have secured a a supply of celluloid scraps from which a far superior solution may be made.

